# Immune-mediated inflammatory diseases and nutrition: results from an online survey on patients’ practices and perceptions

**DOI:** 10.1186/s40795-021-00446-y

**Published:** 2021-07-16

**Authors:** Thao Pham, Harry Sokol, Bruno Halioua, Graziella Pourcel, Manuel Brun, Emilie Pain, Damien Testa

**Affiliations:** 1 Service de rhumatologie, Hôpital Sainte-Marguerite, Aix Marseille Univ, Assistance publique – Hôpitaux de Marseille, Marseille, France; 2 Gastroenterology department, Sorbonne Université, INSERM, Centre de Recherche Saint-Antoine, CRSA, AP-HP, Saint Antoine Hospital, 75012 Paris, France; 3 INRAE, UMR1319 Micalis & AgroParisTech, 78350, Jouy en Josas, France; 4 Dermatologue, Paris, France; 5 Fresenius Kabi, Sevres, Paris, France; 6 Carenity, Communauté de patients en ligne, Paris, France

**Keywords:** Dietary behavior, Immune-mediated inflammatory diseases, Patient’s perception, Online patient community

## Abstract

**Background:**

The central role of microbiota and the contribution of diet in immune-mediated inflammatory diseases (IMID) are increasingly examined. However, patients’ perspectives on nutrition and its impact on their disease has not received a lot of attention. We aimed to directly collect information from patients with IMID about their dietary behaviors and their perceptions of the influence of nutrition on their disease.

**Methods:**

Adult patients with rheumatoid arthritis, ankylosing spondylitis, psoriatic arthritis, Crohn’s disease, ulcerative colitis or psoriasis registered in an online patient community were invited to participate in the study and complete an online self-administered questionnaire. We assessed patients’ dietary knowledge and choices by collecting information on the diet regimens they were following or recommended and their perceptions of the diet and its consequences on their disease.

**Results:**

Fifty patients per target disease were included with a mean age of 48.1 years (95%CI 46.7–49.6). Other sociodemographic and clinical characteristics varied across the diseases. Since diagnosis, 44% of the patients changed their eating habits, mainly patients with inflammatory bowel disease with 69% of these making the change on their own initiative. Patients who did not change their diet habits reported not having received nutritional advice from their healthcare professionals (HCP) in 69% of the cases. The perceived impact of nutrition on their symptoms was mixed (overall 74% of the patients reported positive consequences and 60% negative ones) and varied across the diseases. Patients with psoriasis only experienced positive consequences from changing their diet, such as reduction of stress and improved mental health, while patients with Crohn’s disease reported more negative effects such as increased fatigue and disturbed sleep. Patients with rheumatic diseases and ulcerative colitis reported weight loss and better physical fitness, but also increased fatigue.

**Conclusions:**

Even if differences exist across diseases, the importance of nutrition and its potential positive role in symptom management is acknowledged by the majority of the patients. However, there is a need and a demand from patients to receive more dietary advice. Developing therapeutic education tools on nutrition for people with IMID and involving patients’ organizations would provide useful information and encourage communication between HCP and patients.

## Background

Immune-mediated inflammatory diseases (IMID) cover a wide range of heterogeneous diseases which may affect one specific or multiple organs, tissues or systems [1]. Rheumatoid arthritis (RA), psoriasis (PsO), and chronic inflammatory bowel disease (IBD), which includes Crohn’s disease (CD) and ulcerative colitis (UC), are among the most common IMID [2]. Common characteristic of IMID is their considerable impact on patient’s physical, mental and social well-being and the quality of life of patients and their caregivers [3, 4]. This burden is substantial due to the chronic nature of IMID and because most of them are currently incurable and require lifelong medical treatment. It has also been established that patients with an IMID are at higher risk for developing other IMIDs [5].

While the exact causes of immune system imbalance remain unknown, the coexistence of environmental and genetic factors are suspected to play an important role in the development of IMID. Exposure to environmental factors such as tobacco smoke and other chemicals, infectious agents, pharmaceutical agents, ultraviolet light or socioeconomic status is now commonly known to influence the development of IMID [6–9]. While clinicians’ improved awareness of and diagnosis of IMID may have contributed to the higher global incidence rates of IMID, the significant increase over time paired with regional differences in incidence strongly suggest the influence of environmental factors [10, 11].

Recent evidence suggests that the gut microbiota plays a major role in immune function [12–15] and in IMID [14]. The bacterial composition of the gut microbiome is influenced by many factors including diet, hygiene and sanitary diseases, and smoking or stress [12, 17–16]. There is epidemiological evidence supporting the diet’s role in influencing the gut microbiome with unbalanced gut microbiota composition associated to IMID [18–21].

There is a growing body of research on the contribution of diet in IMID. An association between dietary behavior and the risk of developing an IBD has been shown in several observational studies with an increased risk of CD observed with diet high in fat and meat while high fiber and fruit intakes were associated with decreased risk of CD [22, 23]. Several epidemiological studies suggest that polyunsaturated fatty acids may have a positive effect on RA development or the course of multiple sclerosis through their anti-inflammatory and antioxidant properties [24, 25]. In this context, even if nutritional care appears promising in controlling inflammation in IMID, current nutritional advice in the management of IMID is extremely diverse and varies according to the type of disease [13]. Dietary interventions in IMID management are rarely based on solid scientific background and every disease is not uniformly studied. While some dietary guidelines for IBD are available, less information is available for RA or PsO [26–28]. Enteral nutrition has however demonstrated a positive effect in CD, especially in pediatrics, on controlling disease activity and maintaining remission [29]. Its potent anti-inflammatory effect is well-established and international medical societies recommend enteral nutrition in their guidelines for clinical management of IBD [30, 31]. Moreover, long-term restrictive diets may be harmful to the patient, leading to deficiencies and other clinical consequences, thus the careful monitoring and assessment by healthcare professionals (HCP) including nutritional specialists is advised [15].

While numerous publications highlight the central role of microbiota and the possible impact of some types of diets on some IMID, few studies have evaluated patients’ perception on nutrition and its impact on their disease. As patients’ active involvement in treatment, in general, and dietary therapy, in particular, are key factors for success, we conducted a study to assess patients’ knowledge of and behaviors toward diet and their perceptions of the influence of nutrition and diet on their rheumatologic, gastrointestinal or dermatologic IMID disease.

## Methods

### Study design and participants

We conducted a cross-sectional study through the French Carenity platform (https://www.carenity.com/). Created in 2011, Carenity is an online patient community in which both patients living with a chronic disease and their caregivers can share their experiences, support each other and receive health information. The community also contributes to enhanced medical knowledge by generating real-world patient insights through online surveys which subscribers regularly voluntarily participate in without receiving payment. As of April 2019, when this research project was conducted, more than 500,000 patients and caregivers from six countries (France, Italy, Germany, Spain, United Kingdom and United States of America) were registered in the Carenity platform. In the current study, only patients aged 18 years and older, registered in the Carenity platform, living in France, had self-reported RA, ankylosing spondylitis (AS), psoriatic arthritis (PsA), CD, UC or PsO and consented to participate were eligible to take part in the study and fill out an online self-administered questionnaire. Our initial objective was to recruit 50 respondents per target disease (once the objective was outreached, additional completed questionnaires were removed based on the quality of the verbatim).

### Data collected

A specific self-administered questionnaire was designed for this study to be administered electronically to all patients. It consisted of 36 mandatory questions, 32 closed and 4 open (impact on daily life and ways to help manage the impact, source of nutritional advice, expectations in terms of nutrition-related support). The same questionnaire was administered to all patients. However, questions related to complications were tailored to each category of diseases. Before finalization, the questionnaire was reviewed by three medical experts specialized in each of the disease groups (BH, TP, HS). Additionally, a patient from the French Carenity platform with RA reviewed it to assess its clarity and suitability.

Data were collected in a pseudonymous way over a one-month period.

### Sociodemographic and clinical variables

Sociodemographic variables (age, gender, and socio-professional category using the French Classification of Professions and Socioprofessional Categories from the National Institute of Statistics and Economic Studies (INSEE) - The French Classification of Professions and Socioprofessional Categories classifies the population by a combination of profession (or former profession), hierarchical position and status (salaried employee or otherwise)) along with height and weight were recorded and body mass index (BMI) was calculated. Disease duration was defined as the time between IMID diagnosis and inclusion in the study. Age at diagnosis, comorbidities, HCP in charge of follow-up, care facilities visited, complications and previous and current treatments were collected. Patients’ perceptions of their level of control over their disease was determined through an 11-item scale (0 = not at all controlled; 10 = perfectly controlled).

### Dietary assessment

Dietary behavior was assessed by collecting information on which dietary care regimens were recommended to patients (gluten-free, low-sodium, high-fiber diets, etc.), who recommended these nutritional counseling, changes patients made in their dietary habits since the diagnosis of their IMID, and their perceptions of the consequences of this change. Information on nutrition materials and services recommended to patients were also collected and a 0-to-10 rating scale was used to score patient satisfaction with the recommended information tools (0 = not at all; 10 = totally).

Patients’ perception of nutrition was assessed according to two dimensions: attitude toward change in dietary habits and the reported consequences.

### Statistical analysis

Our objective was to include 50 patients per disease. Eligible patients were included in the study when they started filling in the questionnaire. Only data of patients who completed the questionnaire within the study period (12 April 2019–13 May 2019) were analyzed. A selection of the patients included in the analyses was based on the consistency of the answers and on the time of survey completion once the target was exceeded.

Descriptive statistics were applied. Categorical variables were expressed as absolute frequency and percentages. For continuous variables, data were presented as mean (CI95%: Confident interval 95%) if normal distribution was observed and as median and interquartile range if non-normal distribution was observed. Diseases were categorized into rheumatologic diseases (RA, SA and PsA), gastrointestinal or inflammatory bowel diseases (IBD: CD and UC) and PsO. Results are presented by disease group, overall, and by disease (when relevant). Descriptive analyses were performed using Excel® 2013. Statistical analyses were performed using R studio (v3.5.0). For univariate analyses and continuous data, ANOVA was used to test if there were a difference between more than 2 groups, and Student’s t-test was used to identify where the differences were. Chi-square test was used for categorical data.

## Results

### Study population

**Fig. 1:**
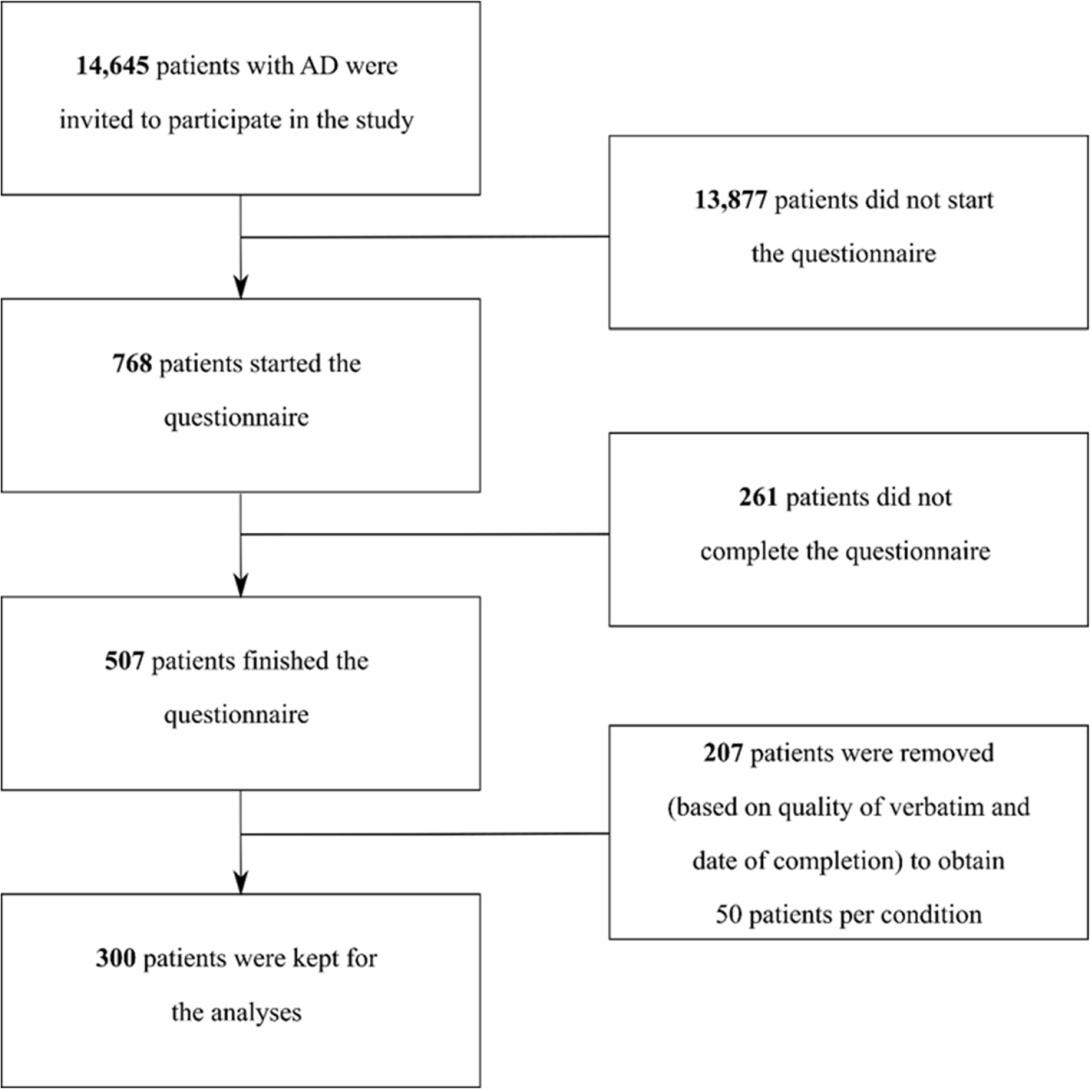
Flowchart of study participants. AD, autoimmune disease

From April 12 to May 13, 2019, 300 patients who fulfilled the inclusion criteria and were willing to participate were included in the study; 50 patients were recruited per target disease (Fig. 1).

The demographic and disease characteristics of the study population are presented in Table 1. While mean age was generally consistent across the diseases (ranging from 44.7 years (95%CI 41.4–48.0) for patients with CD to 50.8 years (95%CI 46.5–55.0) for patients with PsO), some sociodemographic and clinical characteristics were different between the disease populations. The great majority of patients with rheumatologic diseases and CD were women (90% of patients with RA, 88% with AS, 82% with PsA, and 82% with CD), compared to 58% of patients with UC and 68% with PsO (*p* < 0.05). Almost half the overall population were overweight or obese with BMI also varying across diseases: the majority of patients with PsO (54%), RA (60%) and PsA (66%) were overweight or obese compared to patients with IBD (38% of patients with CD and 34% of UC) (*p* < 0.05).

**Table 1:** Socio-demographic and disease characteristics of the study population by disease group (*N* = 300)

Variable	Rheumatic diseases			Gastrointestinal diseases		Psoriasis (*N* = 50)	Overall (N = 300)
	RA (N = 50)	AS (N = 50)	PsA (N = 50)	CD (N = 50)	UC (N = 50)		
**Sociodemographic variables**							
Age, years							
Mean (95%CI)	50.3 (47.0–53.6)	45.7 (42.7–48.7)	50.4 (47.4–53.4)	44.7 (41.4–48.0)	47.0 (42.9–51.1)	50.8 (46.5–55.0)	48.1 (46.7–49.6)
Age groups, N (%)							
18–30 years	3 (6)	4 (8)	2 (4)	6 (12)	6 (12)	4 (8)	25 (8)
31–40 years	8 (16)	13 (26)	7 (14)	12 (24)	12 (24)	12 (24)	64 (21)
41–50 years	13 (26)	14 (28)	17 (34)	17 (34)	10 (20)	11 (22)	82 (28)
51–60 years	17 (34)	15 (30)	15 (30)	9 (18)	14 (28)	8 (16)	78 (26)
61–70 years	8 (16)	3 (6)	8 (16)	6 (12)	5 (10)	9 (18)	39 (13)
> 70 years	1 (2)	1 (2)	1 (2)	0 (0)	3 (6)	6 (12)	12 (4)
Gender, N (%)							
Female	45 (90)	44 (88)	41 (82)	41 (82)	29 (58)	34 (68)	234 (78)
Socio-professional category, N (%)							
Employee	28 (56)	23 (46)	24 (48)	29 (58)	18 (36)	21 (42)	143 (55)
Executive and senior intellectual profession	12 (24)	14 (28)	7 (14)	8 (16)	11 (22)	16 (32)	68 (26)
Intermediate profession	1 (2)	4 (8)	5 (10)	3 (6)	4 (8)	1 (2)	18 (7)
Craftsman, trader, company manager	3 (6)	4 (8)	3 (6)	1 (2)	4 (8)	3 (6)	18 (7)
Worker	1 (2)	0 (0)	2 (4)	3 (6)	4 (8)	4 (8)	14 (5)
Not specified	5 (10)	5 (10)	9 (18)	6 (12)	9 (18)	5 (10)	39 (13)
Body Mass Index, N (%)							
Underweight (< 18.5 kg/m^2^)	4 (8)	4 (8)	2 (4)	5 (10)	4 (8)	3 (6)	22 (7)
Normal weight (18.5 and 25 kg/m^2^)	16 (32)	27 (54)	14 (28)	26 (52)	29 (58)	20 (40)	132 (44)
Overweight (25 and 30 kg/m^2^)	16 (32)	14 (28)	17 (34)	13 (26)	12 (24)	10 (20)	82 (28)
Obese (> 30 kg/m^2^)	14 (28)	5 (10)	14 (28)	6 (12)	5 (10)	17 (34)	61 (20)
Inconsistent answer	0 (0)	0 (0)	3 (6)	0 (0)	0 (0)	0	3 (1)
**Disease variables**							
Disease duration, years							
Mean (95%CI)	8.0 (6.0–10.0)	5.8 (4.2–7.5)	8.0 (4.6–11.4)	15.1 (12.4–17.8)	10.7 (7.7–13.8)	23.5 (19.5–27.4)	11.5 (10.2–12.8)
Age at diagnosis, years							
Mean (95%CI)	42.4 (39.6–45.3)	39.6 (36.3–43.0)	42.0 (38.3–45.5)	29.6 (26.2–32.9)	36.8 (33.4–40.2)	27.0 (22.0–32.0)	36.5 (34.9–38.1)
Comorbidities, N (%)							
Another IMID	11 (22)	33 (66)	24 (48)	22 (44)	18 (36)	22 (44)	130 (43)
HCP, N (%)							
Rheumatologist	47 (94)	47 (94)	46 (92)	5 ()	1 (2)	2 (4)	148 (49)
Gastroenterologist	2 (4)	2 (4)	0 (0)	47 (94)	43 (86)	0 (0)	94 (31)
General practitioner	15 (30)	19 (38)	8 (16)	6 (12)	11 (22)	12 (24)	71 (24)
Dermatologist	1 (2)	3 (6)	8 (16)	3 (6)	3 (6)	28 (56)	46 (15)
Not monitored	1 (2)	1 (2)	0 (0)	2 (4)	6 (12)	11 (22)	21 (7)
Care facilities, N (%)							
Hospital or clinic only	20 (41)	21 (43)	19 (38)	34 (71)	20 (45)	10 (26)	124 (44)
Private practice only	12 (24)	7 (14)	22 (44)	4 (8)	11 (25)	22 (56)	78 (28)
Both private practice and hospital/clinic	17 (35)	21 (43)	9 (18)	10 (21)	13 (30)	7 (18)	77 (28)
Complications, N (%)							
At least one	39 (78)	44 (88)	42 (84)	48 (96)	41 (82)	38 (76)	NA
Depression	15 (30)	22 (44)	22 (44)	23 (46)	19 (38)	20 (40)	NA
Overweight	20 (40)	13 (26)	20 (40)	16 (32)	9 (18)	13 (26)	NA
Appetite disorders	11 (22)	13 (26)	8 (16)	16 (32)	13 (26)	9 (18)	NA
Articular disorders	NC	NC	NC	28 (56)	13 (26)	19 (38)	NA
Dry eyes/mouth	16 (32)	18 (36)	15 (30)	NC	NC	NC	NA
None	11 (22)	6 (12)	8 (16)	2 (4)	9 (18)	12 (24)	NA
**Perception of the patients’ level of control of the disease, score**							
Mean (95%CI)	5.9 (5.2–6.5)	4.5 (3.8–5.1)	4.2 (3.4–5.0)	6.1 (5.4–6.8)	5.7 (4.9–6.5)	4.8 (4.0–5.5)	5.2 (4.9–5.5)
Poorly controlled (score < 4), N (%)	9 (18)	18 (36)	21 (42)	11 (22)	13 (26)	20 (40)	92 (31)
Moderately controlled (score 4–7)	23 (46)	21 (42)	17 (34)	16 (32)	16 (32)	16 (32)	109 (36)
Well controlled (score ≥ 7)	18 (36)	11 (22)	12 (24)	23 (46)	21 (42)	14 (28)	99 (33)

*Abbreviations*: *NC* Not collected, *NA* Not available, *IMID* Immune-mediated inflammatory diseases, *HCP* Healthcare professionals, *AS* Ankylosing spondylitis, *RA* Rheumatoid arthritis, *PsA* Psoriatic arthritis, *CD* Crohn’s disease, *UC* Ulcerative colitis

Age at diagnosis and mean time since diagnosis varied widely across diseases. The mean duration of illness was longest for patients with PsO and they were diagnosed at a younger age than patients with IBD and rheumatic diseases which were diagnosed more recently at a more advanced age. At least four of 10 patients declared having another chronic disease, ranging from 22% of patients with RA to 66% of patients with AS (p < 0.05). In 38% of the cases, the other chronic disease reported was another IMID: PsO and AS were reported in 13 patients (10%), CD in 11 patients (8%), asthma and fibromyalgia both in 8 patients (6%), PsA in 7 patients (5%), and multiple sclerosis in 5 patients (4%). Regardless of the disease, more than three quarters of patients reported complications since the time of their diagnosis of their IMID. Complications were different across diseases, but depression and overweight were reported by approximately four of 10 and more than a quarter of all patients, respectively.

At the time of their inclusion in the study, 93% of all patients were monitored by an HCP. While at least 90% of patients with rheumatologic diseases received care from a rheumatologist (94% with RA, 94% with AS and 92% with PsA) and more than 85% of patients with IBD from a gastroenterologist (94% with CD and 86% with UC), both mainly at a hospital or in a clinic, only 56% of patients with PsO were monitored by a dermatologist and 24% by a general practitioner, mainly in private practice (74%). Noteworthy, 22% of patients with PsO and 12% of patients with UC were not currently under the care of an HCP. Patients with PsA declared attending a hospital or clinic in 56% of the cases.

Patients’ perception of their level of control of their disease varied across disease groups and within a group (Table 1).

**Fig. 2:**
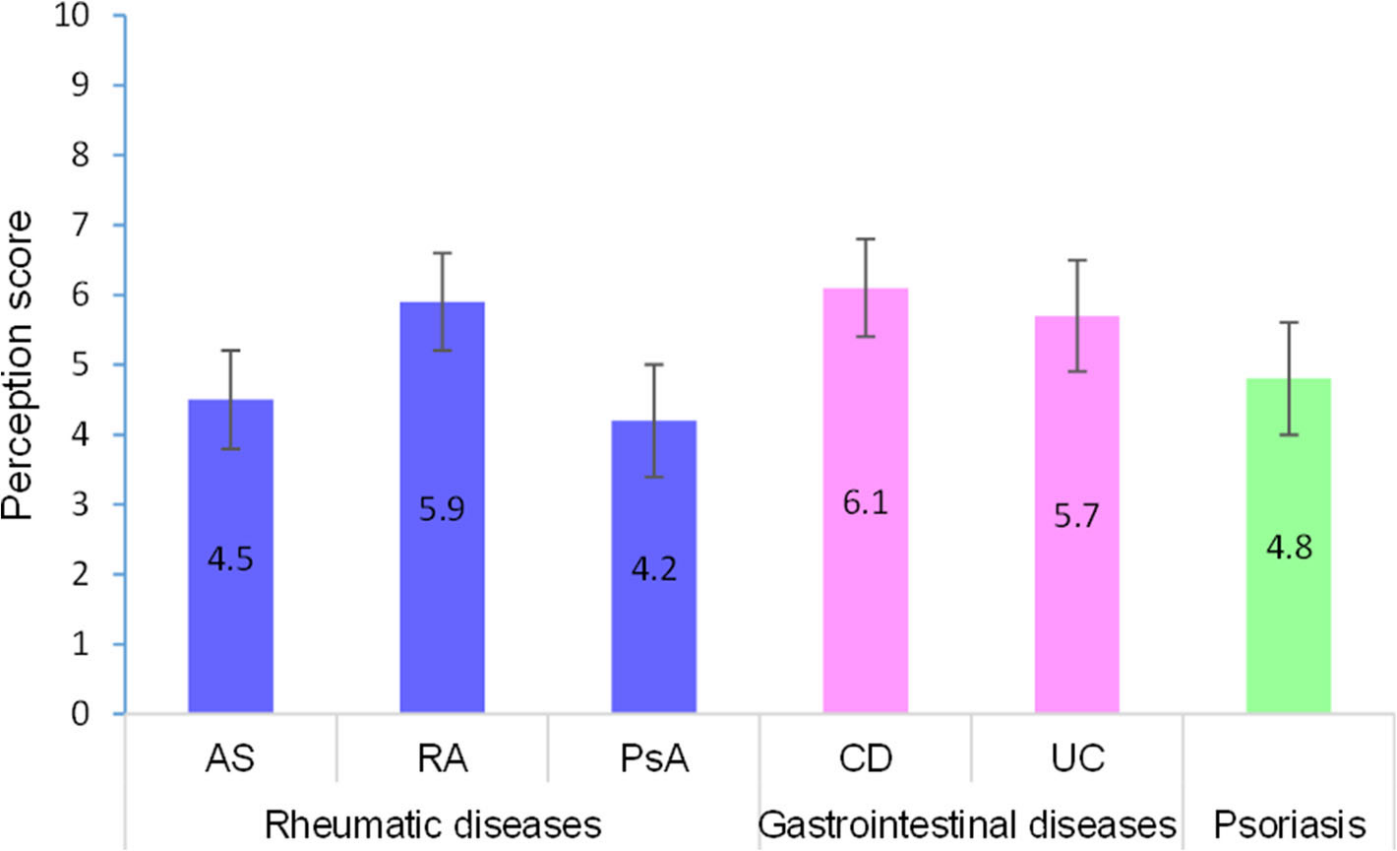
Mean scores of patients’ perception of the level of control of their disease (N = 300). Bars above and below the histogram indicate the upper and lower bounds of the CI95%. Patients perceptions of their level of control over their disease was determined through an 11-item scale (0 = not at all controlled; 10 = perfectly controlled). AS, ankylosing spondylitis; RA, rheumatoid arthritis; PsA, psoriatic arthritis; CD, Crohn’s disease; UC, ulcerative colitis; PsO, psoriasis

Lower level of control was observed for patients with PsA (4.2/10 95%CI 3.4–5.0), AS (4.5/10 95%CI 3.8–5.1) and PsO (4.8/10 95%CI 4.0–5.5). Patients with CD reported a better control of their disease (6.1/10 95%CI 5.4–6.8) (*p* < 0.05) (Fig. 2).

### Nutritional advices

Throughout their care history, more than half of all patients (56%) were given some nutritional advice, mostly by the HCP monitoring their disease (36%) or another HCP (42%). Some patients reported having received recommendations from other patients or the Internet (25%), but very few received information from patient organizations (5%). The type of recommendations varied by the disease (Table 2). The diets recommended for patients with rheumatic diseases varied according to the disease; patients with AS were mainly advised to consume a gluten-free (32%) diet, different diets were recommended to patients with RA and PsA. Low-salt, low-calorie, low-sugar or gluten-free diets were recommended to 38, 30, 26 and 24% of patients with RA, respectively. Patients with PsA were advised to reduce their sugar (26%) and salt (20%) intake or eat a gluten-free (20%) diet. Patients with IBD were more frequently advised to have a low-fiber (58% of patients with CD and 42% with UC), low-salt (34% of patients with CD and 32% with UC) or gluten-free (32% of patients with CD and 32% with UC) diet. Patients with PsO declared having received little advice on nutrition (Table 2). Type of recommendation also varied by patients’ BMI: reducing calorie and sugar intake was frequently advised to overweight patients (62 and 57%, respectively, compared to 38 and 43% for underweight or normal weight patients).

**Table 2:** Type of nutritional advice received by disease (N = 300)

	Rheumatic diseases			Gastrointestinal diseases		Psoriasis (N = 50) N (%)	Overall (N = 300) N (%)
	AS	RA	PsA	CD	UC		
	(N = 50) N (%)	(N = 50) N (%)	(N = 50) N (%)	(N = 50) N (%)	(N = 50) N (%)		
Gluten-free diet	16 (32)	12 (24)	10 (20)	16 (32)	16 (32)	4 (8)	74 (25)
Low-salt diet	4 (8)	19 (38)	10 (20)	17 (34)	16 (32)	2 (4)	68 (23)
Low-fiber diet	4 (8)	5 (10)	3 (6)	29 (58)	21 (42)	0 (0)	62 (21)
Low-sugar diet	4 (8)	13 (26)	13 (26)	10 (20)	12 (24)	8 (16)	60 (20)
High-fiber diet	8 (16)	11 (22)	6 (12)	2 (4)	15 (30)	6 (12)	48 (16)
Low-calorie diet	3 (6)	15 (30)	8 (16)	7 (14)	8 (16)	1 (2)	42 (14)
High-protein diet	4 (8)	8 (16)	4 (8)	12 (24)	11 (22)	2 (4)	41 (14)
Fasting diet	3 (6)	4 (8)	5 (10)	9 (18)	13 (26)	5 (10)	39 (13)
No recommendation	29 (58)	17 (34)	27 (54)	10 (20)	15 (30)	35 (70)	133 (44)

*Abbreviations*: *AS* Ankylosing spondylitis, *RA* Rheumatoid arthritis, *PsA* Psoriatic arthritis, *CD* Crohn’s disease, *UC* Ulcerative colitis

### Change in dietary habits

Since diagnosis, more IBD patients stated they changed their eating habits (80% of patients with CD and 60% with UC) compared to the other patients (42% of patients with RA, 32% with AS, 28% with PsA, and 20% with PsO) (*p* = 0.1) (Table 3). Regardless of the disease, this change was initiated more frequently by the patient than by their HCP (69% vs 31% overall). Of the 169 respondents who did not change their eating habits after initial diagnosis, 69% did not receive nutritional advice from their HCP. Of the patients with rheumatic diseases (50% of patients with RA, 36% with AS and 28% with PsA) and IBD (32% of patients with CD and 34% with UC) who received nutritional advice from their HCPs, between 14 and 69% followed this advice (69% of patients with CD, 59% with UC, 52% with RA, 44% with AS, and 14% with PsA). Only 8 of 50 patients with PsO were recommended specific diets and 2 of them applied them (Table 3).

**Table 3:** Change in eating habits since diagnosis, by disease group (N = 300)

Variable	Rheumatic diseases			Gastrointestinal diseases		Psoriasis (N = 50)	Overall (*N* = 300)
	RA (N = 50)	AS (*N* = 50)	PsA (*N* = 50)	CD (N = 50)	UC (N = 50)		
Change, N (%)							
Yes	21 (42)	16 (32)	14 (28)	40 (80)	30 (60)	10 (20)	131 (44)
No	29 (58)	34 (68)	36 (72)	10 (20)	20 (40)	40 (80)	169 (56)
Person who recommended change, N (%)							
Patient his/herself	8 (38)	8 (50)	12 (86)	29 (73)	20 (67)	8 (80)	90 (69)
HCP	13 (62)	8 (50)	2 (14)	11 (27)	10 (33)	2 (20)	41 (31)
Reason for no change, N (%)							
Patient decision despite HCP advice, %	12 (41)	10 (29)	12 (33)	5 (50)	7 (35)	6 (15)	52 (31)
No advice from HCP, %	17 (59)	24 (71)	24 (67)	5 (50)	13 (65)	34 (85)	117 (69)

*Abbreviations*: *HCP* Healthcare professionals, *AS* Ankylosing spondylitis, *RA* Rheumatoid arthritis, *PsA* Psoriatic arthritis, *CD* Crohn’s disease, *UC* Ulcerative colitis

### Impact of diet change

Two thirds of the patients (66%) who had modified their diet experienced a change as a consequence. Even though positive consequences including weight loss, better physical fitness and improved mental health were observed by 27, 27 and 13% of patients, some negative consequences were reported such as increased fatigue (21%), disturbed sleep (15%) and difficulty carrying out normal physical activities (14%). The perceived consequences of the change in diet varied across the diseases. Patients with rheumatic diseases reported weight loss (44% of patients with AS, 33% with RA, and 21% with PsA) and better physical fitness (36% of patients with PsA, 29% with RA, and 25% with AS) but 24% of those with RA also mentioned increased fatigue. While 43% of patients with UC declared having better physical fitness and few negative effects (13% of all UC cases), patients with CD mentioned increased tiredness (43%), disturbed sleep (28%) and difficulty carrying out normal physical activities (28%). Noteworthy, one of five patients with CD reported feeling their nutritional intake was insufficient. Patients with PsO reported, beyond weight loss (50%), better physical activity (30%), improved mental health (30%) and reduced stress (20%), but no negative consequences. A non-negligible proportion of all patients stated they did not feel the change in their diet produced any consequences (25–38% of patients) (Table 4).

**Table 4:** Consequences of the change in eating habits, by disease group (*N* = 131 who have changed their diet)

Variable	Rheumatic diseases			Gastrointestinal diseases		Psoriasis (*N* = 10)	Overall (N = 131)
	RA (*N* = 21)	AS (*N* = 16)	PsA (*N* = 14)	CD (*N* = 40)	UC (N = 30)		
Consequences of the diet change, N (%)							
Weight loss	7 (33)	7 (44)	3 (21)	8 (19)	6 (20)	5 (50)	36 (27)
Better physical fitness	6 (29)	4 (25)	5 (36)	5 (13)	13 (43)	3 (30)	36 (27)
Increased tiredness	5 (24)	1 (6)	1 (7)	17 (43)	4 (13)	0 (0)	28 (21)
Increased sleep disorders	2 (10)	2 (12)	1 (7)	11 (28)	4 (13)	0 (0)	20 (15)
Difficulties in keeping physical activity	1 (5)	1 (6)	1 (7)	11 (28)	4 (13)	0 (0)	18 (14)
Improved mental health	4 (19)	2 (12)	2 (14)	2 (5)	4 (13)	3 (30)	17 (13)
Undernutrition	1 (5)	0 (0)	0 (0)	9 (23)	3 (10)	0 (0)	13 (10)
Reduced stress	0 (0)	0 (0)	1 (7)	2 (5)	4 (13)	2 (20)	9 (7)
Other	1 (5)	4 (25)	3 (21)	3 (8)	6 (20)	1 (10)	18 (14)
I did not feel any particular change	8 (38)	4 (25)	5 (36)	14 (35)	10 (33)	3 (30)	44 (34)

*Abbreviations*: *AS* Ankylosing spondylitis, *RA* Rheumatoid arthritis, *PsA* Psoriatic arthritis, *CD* Crohn’s disease, *UC* Ulcerative colitis

### Nutrition services and informational materials

Overall, 24% of the patients were offered informational materials or services on nutrition. This varied depending on the diseases: 16% with rheumatic diseases (8% for PsA, 16% for AS and 24% for RA patients), 40% of patients with IBD (40% for both patients with CD and UC), and 10% with PsO (*p* < 0.05). They were mainly offered brochures (29%) or referral to nutritionist services (28%). Overall, patients were slightly dissatisfied with the information and/or services provided (overall median score = 4.5/10, with 10 = totally satisfied; Q1-Q3:2.5–6.0). Median satisfaction scores varied across diseases from 2.5 (Q1-Q3:2.5–5.5) for AS to 4.8 for CD (Q1-Q3:3.1–6.0) to UC (Q1-Q3:2.0–6.4) patients. Patients who had changed their eating habits as per the advice of HCP tended to be more satisfied with nutrition services and informational materials (mean score = 5.8 (95%: 4.6–6.9)) than those who self-imposed their diet (mean score = 3.8 (95%: 2.8–4.8)).

## Discussion

A combination of genetic susceptibility and environmental exposure are suspected to be the cause of IMID [7, 32]. Among the environmental factors, nutrition may play a role in IMID, both through the intestinal microbiota and dietary intake leading to overweight [8]. However, the role of nutrition in disease management from the patients’ perspectives has rarely been described. It is now widely agreed that patients’ involvement in therapeutic care, both pharmacological and non-drug interventions, improves health outcomes. Patients’ active participation and involvement in decisions on their own care process and health is encouraged [33]. In the context of dietary therapy, patients’ active involvement in decision-making and adherence to treatment are key to observing benefits [34]. In fact, the health benefits of dietary treatment is more closely related to the degree of the individual’s adherence to treatment than the type of diet administered [35]. To adequately involve the patient in his/her own care, it is important to acknowledge his/her perceptions of the proposed therapy.

To our knowledge, this is the first observational study to collect real-life data about patients’ attitudes and perceptions of nutrition and its impact on their disease in different types of IMIDs, including rheumatic diseases and PsO. Our findings show that patients consider nutrition an important aspect of their disease management. Almost half the study participants (44%) changed their eating habits, mainly patients with IBD. Of these patients, more than two thirds made the change on their own initiative. However, we don’t know if the dietary adjustments made by the patients were aligned with the advices given by HCPs. Among the patients who did not change their eating habits, 69% stated they did not receive nutritional advice from their HCP. The results also suggest patients positively perceived the impact of nutrition on their symptoms, with 74% reporting positive consequences; however, 60% of patients reported some negative consequences of their dietary changes, with consequences varying by disease. The 10 patients with PsO who made dietary changes only experienced positive consequences including reduction of stress and improved mental health which is particularly beneficial to these patients as they are commonly affected by stress and anxiety. Patients with rheumatic diseases and UC reported positive impacts such as weight loss and better physical fitness, but also increased fatigue. Even if some of them reported weight loss and better physical fitness, patients with CD tended to report rather negative effects such as increased fatigue, disturbed sleep and difficulty carrying out normal physical activities.

Half the patients who felt their disease was well controlled (perception score > 7) had changed their eating habits, against 43 and 38% of patients who felt their disease was moderately and poorly controlled, respectively. Only one quarter of the patients who received nutrition services or informational materials in the form of brochures or access to a nutritionist were moderately satisfied with those tools. The majority of those patients reported with IBD. Regardless whether patients have already received dietary advice, half of them voluntarily mentioned they had additional expectations about nutrition-related information, mainly practical, general, or disease-specific advice, to be given by nutritionists or clinicians. This information gap led them to independently research and implement dietary interventions from other sources such as the internet without professional monitoring. This may produce suboptimal disease management, worsen the disease, create deficiencies, facilitate the development of other diseases, and may even be dangerous in the case of restrictive or popular fad diets. Additionally, some diets are associated with a non-negligible burden for the patients which can impair their quality of life and lead to the discontinuation of therapy.

As seen above, the potential negative side effects of diets combined with the high proportion of patients who are self-initiating dietary changes strongly suggests the importance of carefully-advised nutritional intervention and monitoring by professionals: the HCP in charge of the patient and nutrition specialists. Effective communication and collaboration between patients and HCPs is known to increase patient adherence [36] and helps HCPs to understand patients’ difficulties, perspectives and perceptions while informing their jointly-planned treatment course. While patients’ organizations are very active in nutrition in some diseases, especially IBD, it’s surprising that very few patients from our survey declared having received dietary recommendations from them. There is, thus, still room for increasing patient organizations’ involvement in promoting nutritional interventions in collaboration with HCP.

The study population was recruited from a patient social platform which has several benefits. Firstly, patients in social networks are more willing to share experience and feel freer to express themselves anonymously and confidentially without their HCP’s involvement (which has been shown to limit the social desirability bias [37]). Secondly, those networks provide an opportunity to collect patient-reported outcomes that complement and add value to clinical data while empowering patients and putting them at the center of their own care. The use of such patients’ platforms; however, limits the researcher’s ability to include individuals who do not have access to the online tool and thus may lead to a potential recruitment bias [38].

In our study, all data were reported by the patients themselves through closed-ended but also open-ended questions, which allowed for collection of patient opinions, perceptions and expectations; however, clinical data provided by the patient was not confirmed by clinicians, thus introducing self-reporting bias including recall bias. Furthermore, as is common in self-administered questionnaires, only patients having the capacity to answer have participated. In addition, as the objective was to reach 50 complete questionnaires per condition in order to be able to compare the different conditions studied and this objective was outreached in some of the conditions, 207 completed questionnaires were removed based on the quality of the verbatim which could lead to a selection bias.

## Conclusion

Even if differences exist across diseases, a similar behavior towards a positive perception of the importance of nutrition and its potential role in symptoms management was reported by most of the IMID patients. While the role played by diet in inflammatory process is more and more considered and scientific and medical data start emerging for some diseases, robust data coming from well-designed clinical trials are still insufficient to allow evidence-based nutritional recommendations and systematic integration into the normal comprehensive care pathway [21]. However, as highlighted in our study, there is a great need and demand from patients to receive dietary advices from their HCP. This suggests that developing therapeutic education tools on nutrition for people affected by IMID would provide useful information to the patient and his/her family and relatives even if it may not completely meet patients’ expectations for nutrition education. It will, however, encourage effective communication between HCP and patients with IMID which a key component of patients care. Patients’ organizations involvement in dietary recommendations as a therapeutic measure in IMID management may also accentuate patient satisfaction.

## Data Availability

The datasets used and/or analyzed during the current study are available from the corresponding author on reasonable request.

## Abbreviations

AS: Ankylosing spondylitis
BMI: Body mass index
CD: Crohn’s disease
CI95%: Confident interval 95%
HCP: Healthcare professional
IBD: Inflammatory Bowel Disease
IMID: Immune-mediated inflammatory diseases
PsA: Psoriatic arthritis
PsO: Psoriasis
RA: Rheumatoid Arthritis
UC: Ulcerative colitis

## References

[1] American Autoimmune Related Diseases Association. Autoimmune diseases list. Available from: https://www.aarda.org/diseaselist/. Accessed Oct 2019.

[2] Meccain J. The Disease Burden of the Most Common Autoimmune Diseases. Manag Care 2016; 25(7): 28–32. Available from: https://www.managedcaremag.com/archives/2016/7/disease-burden-most-common-autoimmune-diseases. Accessed Oct 2019. 28121529

[3] Olesińska M, Saletra A (2018). Quality of life in systemic lupus erythematosus and its measurement. Reumatologia.

[4] Greenfield J, Hudson M, Vinet E, Fortin PR, Bykerk V, Pineau CA, Canadian Scleroderma Research Group and Canadian Inflammatory Myopathy Study Group (2017). A comparison of health-related quality of life (HRQoL) across four systemic autoimmune rheumatic diseases (SARDs). PLoS One.

[5] Makredes M, Robinson D, Bala M, Kimball AB (2009). The burden of autoimmune disease: a comparison of prevalence ratios in patients with psoriatic arthritis and psoriasis. J Am Acad Dermatol.

[6] Wang L, Wang FS, Gershwin ME (2015). Human autoimmune diseases: a comprehensive update. J Intern Med.

[7] Anaya JM (2010). The autoimmune tautology. Arthritis res Ther.

[8] Anaya JM, Ramirez-Santana C, Alzate MA, Molano-Gonzalez N, Rojas-Villarraga A (2016). The Autoimmune Ecology. Front Immunol.

[9] Brodin P, Jojic V, Gao T, Bhattacharya S, Angel CJ, Furman D (2015). Variation in the human immune system is largely driven by non-heritable influences. Cell.

[10] Schmidt CW (2011). Questions persist: environmental factors in autoimmune disease. Environ Health Perspect.

[11] Thorburn AN, Macia L, Mackay CR (2014). Diet, metabolites, and “western-lifestyle” inflammatory diseases. Immunity.

[12] Vieira SM, Pagovich OE, Kriegel MA (2014). Diet, microbiota and autoimmune diseases. Lupus.

[13] Choi IY, Lee C, Longo VD (2017). Nutrition and fasting mimicking diets in the prevention and treatment of autoimmune diseases and immunosenescence. Mol Cell Endocrinol.

[14] Masuko K (2018). A Potential Benefit of “Balanced Diet” for Rheumatoid Arthritis. Front Med.

[15] European Parliament (2017). Autoimmune diseases – modern diseases: report.

[16] Scudellari M (2017). Cleaning up the hygiene hypothesis. Proc Natl Acad Sci.

[17] Sharpton T, Lyalina S, Luong J, Pham J, Deal EM, Armour C (2017). Development of inflammatory bowel disease is linked to a longitudinal restructuring of the gut metagenome in mice. mSystems.

[18] Knoll RL, Forslund K, Kultima JR, Meyer CU, Kullmer U, Sunagawa S (2017). Gut microbiota differs between children with inflammatory bowel disease and healthy siblings in taxonomic and functional composition: a metagenomic analysis. Am J Physiol-Gastrointest Liver Physiol.

[19] Liu X, Zeng B, Zhang J, Li W, Mou F, Wang H (2016). Role of the gut microbiome in modulating arthritis progression in mice. Sci Rep.

[20] Chen J, Wright K, Davis JM, Jeraldo P, Marietta EV, Murray J (2016). An expansion of rare lineage intestinal microbes characterizes rheumatoid arthritis. Genome Med.

[21] Lee D, Albenberg L, Compher C, Baldassano R, Piccoli D, Lewis JD (2015). Diet in the Pathogenesis and Treatment of Inflammatory Bowel Diseases. Gastroenterology.

[22] Hou JK, Abraham B, El-Serag H (2011). Dietary intake and risk of developing inflammatory bowel disease: a systematic review of the literature. Am J Gastroenterol.

[23] Gioia C, Lucchino B, Tarsitano MG, Iannuccelli C, Di Franco M (2020). Dietary Habits and Nutrition in Rheumatoid Arthritis: Can Diet Influence Disease Development and Clinical Manifestations?. Nutrients.

[24] Schwarz S, Leweling H (2005). Multiple sclerosis and nutrition. Mult Scler.

[25] Brown AC, Rampertab SD, Mullin GE (2011). Existing dietary guidelines for Crohn's disease and ulcerative colitis. Expert Rev Gastroenterol Hepatol.

[26] Durchschein F, Petritsch W, Hammer HF (2016). Diet therapy for inflammatory bowel diseases: the established and the new. World J Gastroenterol.

[27] Vitetta L, Coulson S, Schloss B, Allen SA (2012). Dietary recommendations for patients with rheumatoid arthritis: a review. Nutrition and Dietary Supplements. Nutr Diet Suppl.

[28] Hansen T, Duerksen DR. Enteral Nutrition in the Management of Pediatric and Adult Crohn's Disease. Nutrients. 2018;10(5). 10.3390/nu10050537. 10.3390/nu10050537 PMC5986417 29701656

[29] Comeche JM, Caballero P, Gutierrez-Hervas A (2019). Enteral Nutrition in Patients with Inflammatory Bowel Disease. Systematic Review, Meta-Analysis, and Meta-Regression. Nutrients.

[30] Ruemmele FM, Veres G, Kolho KL, Griffiths A, Levine A, Escher JC, Dias JA, Barabino A, Braegger CP, Bronsky J, Buderus S, Martín-de-Carpi J, De Ridder L, Fagerberg UL, Hugot JP, Kierkus J, Kolacek S, Koletzko S, Lionetti P, Miele E, López VMN, Paerregaard A, Russell RK, Serban DE, Shaoul R, Van Rheenen P, Veereman G, Weiss B, Wilson D, Dignass A, Eliakim A, Winter H, Turner D (2014). Consensus guidelines of ECCO/ESPGHAN on the medical management of pediatric Crohn's disease. J Crohns Colitis.

[31] Cárdenas-Roldán J, Rojas-Villarraga A, Anaya JM (2013). How do autoimmune diseases cluster in families? A systematic review and meta-analysis. BMC Med.

[32] Thompson AG (2007). The meaning of patient involvement and participation in health care consultations: a taxonomy. Soc Sci Med.

[33] Sofi F, Cesari F, Abbate R, Gensini GF, Casini A (2008). Adherence to Mediterranean diet and health status: meta-analysis. Br Med J.

[34] Vaillancourt H, Légaré F, Lapointe A, Deschênes SM, Desroches S (2014). Assessing patients’ involvement in decision making during the nutritional consultation with a dietitian. Health Expect.

[35] Cohen SM (2009). Concept analysis of adherence in the context of cardiovascular risk reduction. Nurs Forum.

[36] Wicks P, Stamford J, Grootenhuis MA, Haverman L, Ahmed S (2014). Innovations in e-health. Qual Life Res.

[37] Ravoire S, Lang M, Perrin E (2017). Advantages and limitations of online communities of patients for research on health products. Therapie.

[38] Lerner A, Jeremias P, Matthias T (2015). The world incidence and prevalence of autoimmune diseases is increasing. Int J Celiac Dis.

